# The idea of society: the Spoken World Theory and the ontological conceptualization of society

**DOI:** 10.3389/fsoc.2023.1241355

**Published:** 2023-10-27

**Authors:** Luk Van Langenhove

**Affiliations:** Brussels School of Governance, Vrije Universiteit Brussel, Ixelles, Belgium

**Keywords:** social theory, ontology, spoken world, Rom Harré, Vygotsky

## Abstract

This article presents a new conceptualization of society with the ambition to sharpen thinking about social reality and to better understand how society relates to personhood. This exercise is framed in an attempt to develop the Spoken World Theory, inspired by the thinking of Rom Harré. It involves a radical rethink of the social ontology and is to be seen as an alternative to the traditional conceptualization of society as a social structure that is opposed to individual agency. The proposed alternative is based upon the disentanglement of four aspects of society along the Vygotskian public/private and individual/collective axes. As such, society can be said to manifest itself in four realms: (i) the *world as we hear* it: a worldwide and history-long ongoing web of conversations; (ii) the *world as we see* it: a set of materialized social artifacts, including a set of institutional facts; (iii) the *world as we imagine* it: individual umwelts or worldviews for each person based on appropriated knowledge and moral frameworks; and (iv) the *world as we* shape it: persons have the power to formulate intentions that they can bring to the conversational space or the space of artifacts. A major consequence of this conceptualization is that it no longer puts society outside human beings, nor that personality is only to be located inside persons. The proposed ontological framework allows us to speak in much clearer terms about how persons and society are entangled with each other in the sense that without the personhood of people, there can be no society, and that without society, people cannot have personhood. Both personhood and society are to be seen as two intertwined mechanisms that allow the individuals of the human species to complement the genetic basis of survival with a system of cultural resources that can be used for coping with everyday life. The article ends with a discussion of the practical implications of social theorizing.

## Introduction

This article introduces a new conceptualization of society based upon a radical rethinking of the ontology of the social realm. It is the ambition of this exercise in theorizing, that it contributes to a clearer understanding of what a society is and how society relates to the existence of personhood at the level of individuals. This entails using a rather complex conceptual apparatus. But as noted by Boyer (2018), there is indeed no good reason “why human societies should not be described and explained with the same precision and success as the rest of nature” (p. 1). However, when looking at the available definitions of society, it becomes rapidly clear that often there is not much conceptual sharpness deployed for describing what a society is. Wikipedia for instance, defines a society as “a group of individuals involved in persistent social interaction, or a large social group sharing the same spatial or social territory, typically subject to the same political authority and dominant cultural expectations.”^1^ If one turns to the academic literature, traditionally, the answers to the question are formulated from different disciplinary perspectives. History gives a diachronical account, anthropology boils it down to divisions of labor and the formation of groups. Sociology stresses the role of so-called structures or emphasizes the constructivist nature of the social realm. Psychology focuses on the development of cognitive capacities and the role of language as an advanced form of communication. The economy stresses the importance of money and the exchange of goods. Most philosophical definitions go in the same direction. Mario Bunge, for example, defines society as a “system of systems—families, firms, schools, states and so on” (Bunge, 1999, p. 61). Very often society is seen as a synonym for social structure that can be contrasted to agency. For Anthony Giddens, this contradiction is a false one, as the human agency and social structure are not to be seen as separate processes but a two-way structuration. Another take comes from Nicolas Luhmann who wrote that society is communication: “there are no other elements: there is no further substance but communications. The society is not built out of human bodies and minds. It is simply “a network of communication” (Luhmann, 2020, p. 100). A somewhat similar approach is found in Zohar and Marshall (1994, p. 23) who think about society as being the domain in which we dwell together with others. It includes anything from intimate relationships to the global world of politics and economics.

One of the most detailed attempts available to formulate an answer to the “what is society?” question, can be found in Babbie (1994) who concludes that society is “a self-structuring, self-organizing, self-creating process” (p. 13). In his book, Babbie defends the case that society is the “unusual entity” of social structure, something that is made out of the freedom of individuals. It makes him say: “surrendered freedom is the substance of society” (p. 46). Notwithstanding the merits of Babbie's approach, he too fails to explain in detail how social structure concretely operates. To be sure, there are other attempts to be found, but as Heintz (2004, p. 24) rightly noticed, the usage of concepts such as “systems” or “emergence” is in general always rather sloppy and inexact. Similarly, Dave Elder-Vass lamented that “one of the problems of the social sciences is a lack of ontological rigor” (Elder-Vass, 2007, p. 2. 228)

Therefore, this random query already points to different approaches to capture the notion of society: systems, structures, freedom, and communication are candidates for being the substance of society. For sure there are many other viewpoints, but regardless of their differences, all these approaches seem to have a single idea in common, namely: “that society is more than the sum of individuals” (Wan, 2011, p. 2). But that claim too is unfortunately not backed by precise conceptualizations. At best, reference is made to the micro–macro distinction or the structure/agency distinction. A lot has been written about the relationship between (societal) structure and (personal) agency, but perhaps Guy (2019) is right when saying that the problem is that one “cannot emphasize one without casting doubts on the other” (p. 26). Mainly because of the ninenteeth century institutionalization of the social sciences in disciplines, the advocates of the agency are to be found in psychology departments, while the aficionados of the structure are in the sociology departments. As Wendt (2015, p. 23) remarks: “many social scientists are not interested in what goes on inside heads, but in the structures that constitute macro-level social systems.” He continues by stating that on top of that duality comes the issue that structures are invisible. In some way, it looks like the concept of society is often reified as a thinglike entity (Hull, 2013, p. 54; Wendt, 2015, p. 25). The big issue then is: if society is not to be regarded as a “thing,” how should it be conceived?

Finding out why society exists, is the necessary first step in theorizing the ontology of society. This needs to be done in such a way that it accounts for the biological and non-biological reasons why society exists. The exercise in theorizing presented below aims therefore to propose a more rigorous conceptualization of the social ontology that takes into account the biological basis of social behavior. It starts from three related ontological questions: (i) Why do societies exist? (ii) How do people make a society operate and function? and (iii) What are the hidden mechanisms that allow societies to sustain and/or change? This article seeks to formulate coherent answers to the abovementioned ontological questions as part of the so-called *Spoken World Theory* (hereafter abbreviated to SWT). After briefly explaining what the intellectual origins and essence of SWT are, attention will go to scrutinizing the assumptions upon which the SWT is based and presenting a new specific part of the theory, namely a new look at the elements that constitute a society.

## The essence and assumptions of the Spoken World Theory

Given the rather poor status of thinking about the essence of society, it should not be a surprise that there is nothing similar in social sciences to what in the natural sciences is called a “standard model” or a “theory of everything.” The natural sciences have indeed since long worked toward synthesizing its results into such a grand theoretical scheme that aims not to predict things but to understand the deep structure of the fabric of reality. The social sciences seem not to have such a tradition to synthesize its results at such an abstract level, let alone integrate insights from different fields or disciplines. As a result, there are many popular books available that explain the “fabric of reality,”^2^ while similar attempts to explain the fabric of social reality are scarce and do not make it to the airport bookshops. As observed by Wendt (2015, p. 1), the main reason for that lacune is that “in contrast to physical sciences (...), where there is broad agreement of the nature of reality and how we should study it, in the social sciences there is no such consensus.” Behind this lack of consensus lies a lot of confusion about the nature of the social realm.

There is of course the academic field of social theory. There is certainly no shortage of books that reproduce over and over the Grand Old Theories, mostly at the undergraduate level. It is striking that less seems to be done on the development of new theories. As a result, the same theories are over and over dealt with. But outside of the social theory community, they are hardly used. Unger (1988) in his book *Social Theory: Its Situation and Its Task* shows the unrecognized transformative possibility, because as testified in many critical accounts, the practical impact of social theory is very limited nowadays. Ever since Goulder (1970), there have been repeated warnings that social theory is in bad shape and there have been equally repeated calls for “integration” between disciplines and within research traditions. An exception to this might be the academic study done on social ontology, for instance, by Tuomela (2013, p. 13), who defines social ontology as “the study of the constituents and construction of social reality.” The issue of social ontology has already been prominently present in the writings of classical thinkers such as Weber of Durkheim, Montesquieu, Tocqueville, and many others. But, as noted by Boyer (2018, p. 1): “there was little sense of cumulative progress.” As a result, and also because of the influence of positivism on the social sciences, interest in a general theory of society seems to have faded away and social ontology became equated with the notion of collective intentionality. Today, social ontology studies hardly contribute to developing syntheses of the knowledge that is available on social reality. It prompted Wan to call for a “re-ontologization” of social reality (Wan, 2011, p. 15).

### The intellectual sources of influence of the Spoken World Theory

Van Langenhove (2021) proposed to use the label “the Spoken World Theory” to refer to a “re-ontologization” effort that can be found in the writings of Harré (1987, 1988), whose attempt to formulate an integrated program for studying psychological and social phenomena is centered around his claim that conversations are key to understanding the functioning of the social world. The related theoretical perspective that will be presented below builds on the studies by Harré and several scholars, who each made conceptual and theoretical progress that can be integrated into the quest to formulate a coherent view of the ontology of the social realm. One can distinguish between two circles of intellectual influence that are shaping the SWT. The first circle consists of the builders of the theory. That includes first of all, Rom Harré who throughout his whole oeuvre developed a strong social constructionist perspective on the social realm, blended with a realist stance.^3^ Especially relevant to the topic considered here is Harré (1998, 2002). Furthermore, there is the study by Van Langenhove (2019, 2020, 2021) who started the attempt to systematize Harré's study. The first circle also includes a group of authors who at some point collaborated with Harré attempted and/or brought several aspects of Harré's study together in a coherent theoretical framework (a non-exhaustive sample should include Bo Christensen, Ali Moghadam, Peter Muhlhauser, Gerrod Parrott, and Grant Gillet). Next, there is a second circle of social theorists and philosophers whose study has inspired Harré and his co-authors. As for social theory, there are plenty of contemporary scholars who addressed general questions and presented comprehensive views on social ontology and the nature of society. Among them are the writings of Anthony Giddens, Jurgen Habermas, John Searle, Ulrich Beck, Ludwig Wittgenstein, and many others, including the philosophers' insights on the advanced ideas of how a society functions or on how a society ideally should look. Classical examples here are Plato and other Greek philosophers who formulated normative ideas on how a state should function.

The SWT can be regarded as an attempt to formulate a coherent view of what society is, based on the premise that conversations are the key to describing and explaining societal processes and that speech-acts in conversations are the real substance of all social and psychological events. In that way, it can be said that humanity lives in a double—interconnected—reality: there is the material world of objects situated in a Euclidian time and space grid and there is the spoken world of speech-acts situated in a non-Euclidian grid of conversations and positioned persons. As will be explained below, conversations, things, and persons are the constituents of society, together with the fields of influence that emerge out of these three entities. The most important fields are moral orders, knowledge about social reality which forms personalized umwelts, and the agency that can be attributed to persons. These aspects will be further discussed below, but first something more about the implicit assumptions of SWT.

### The assumptions of the Spoken World Theory

The Spoken World Theory is as all social theories based on a number of assumptions that can be regarded as postulates. The first assumption of SWT is that because the biological evolution of humans transformed into cultural evolutions, there emerged human beings with personality at the individual level and sociality at the group level.^4^ The second assumption states that the existence of society cannot be understood independently of an understanding of why human beings have a personality and vice versa. The third assumption holds that the understanding of society and personality are best modeled on conversations as it is within conversations that persons and society are constructed through speech-acts.

#### Assumption one: the emergence of society needs to be analyzed from an evolutionary perspective

The first assumption implies that understanding society needs a biological grounding to clarify the linkages between the biological and the social realms of people (Christakis, 2019). Human beings are biological entities that evolved as a species of animals according to the Darwinian principle of natural selection. The existence of society can therefore not be uncoupled from the genetic basis of human beings. To some extent, the daily functioning of a human being depends on genetic predispositions, while to some other extent, it is determined by cultural aspects. This is reflected in the classical nature/nurture debate. One of the crucial differences between nature and nurture is that in the former, the bulk of information to survive is to speak that is stored “inside” an individual as it resides in its genetic material. As for the latter, the information is “outside” the individual, meaning that it resides in culture. The issue, therefore, is not only to determine the proportion between nature and nurture but to know how the information is stored outside the body and how it finds its way to the individuals who need it along with knowing how that information is transferred over the generations. Such a shift from genes toward culture needs mechanisms to link the individual to culture. This seems to be a crucial aspect of any social theory of society: its ability to explain how individuals (with genetic characteristics) and culture (that is created and appropriated by these individuals) can co-exist and interact with each other. Moreover, this implies that there needs to be mechanisms to keep the information available across generations and make the organism use it. It is there that lies the origin of the emergence of societies and personality. The information to survive must be transferable over the generations, which implies that it needs to be stored independently of individual mortal persons.

To summarize: human beings have developed two mechanisms that allow the evolutionary transformation from a biological level to a cultural level: personality and sociality.^5^ If the information necessary to survive is stored outside the organism, then not genes but culture becomes the source and driver of evolution. The core problem to address is how the present and past organization of the human species fits within the evolutionary development of the hominids.

#### Assumption two: persons and societies are so entangled with each other that one cannot be studied without the other

Two things stand out if one compares humans with other species of animals: the existence of human personality and the fact that humans “build complex and apparently different societies” (Boyer, 2018, p. ix). Of course, one can observe that there are lots of animal species that also have some kind of social life. They work together, they live together, and biologists such as Ward (2020) even claim that some animals actually also build societies. While it cannot be denied that cooperation varies widely across species, there is, however, no animal that has a form of society that is even remotely as complex as human society. On top, societies have a history: they change and people are capable of reflecting upon that history and are able to pass knowledge down through generations. Nothing in the animal world compares to that. Nevertheless, human beings are animals too, and thus biological creatures. This suggests that there must have been evolutionary developments that led to the emergence of human society. The same holds for the cognitive capacities of human beings: people have developed extraordinary cognitive capacities, including language, and the ability to operate in groups, while at the same time having a strong sense of individual identity as reflected in personality. Again, there are some animal species where individual differences can be observed and perhaps even personality traits, and for sure the cognitive capacities of animals such as octopuses are not to be underestimated (Godfrey-Smith, 2016). But still, there is no other animal species on the planet that has a knowledge system that allows them to process food, build particle accelerators and castles, and so much more. All of this leads to the conclusion that the existence of society and the presence of personhood is what makes the human species special. Are personhood and society two independent developments of evolutionary processes or is there a relationship between both concepts that makes it no coincidence that both are so characteristic of humanity?

Although it seems to make sense to study the origins of personhood and society together, this is to the best of my knowledge hardly happening. Looking for answers to the above questions is not favored by the disciplinary divides in the social sciences. Matthews (2020) defines this event as a grand challenge for personality psychology and social psychology. Others have voiced similar concerns. As a result, knowledge about the relationship between the individual and society is very fragmented. Sociology and psychology developed as independent disciplines, meaning not only that they have different subject matters but also different professional organizations (Manicas, 1987). Today, we are witnessing a watershed between scholarly interest for either the individual or the collectivity, notwithstanding all calls for interdisciplinarity. The paradox is that increased interactions between disciplines can lead to institutionalized interactions that even can take the form of new disciplines. As such, attempts to link sociology to psychology have resulted in so-called “social psychology.” But then, when social psychology became institutionalized as well, it became regarded as a “level” between the micro and the macro levels. In that way, the social world became modeled after the disciplinary divides in the natural sciences. For the natural realm, a distinction is made among the study of quantum physical reality, the study of atoms (physics), the study of molecules (chemistry), and the study of objects (mechanics). Moreover, because the boundaries between the disciplines are sometimes fuzzy, there are intermediate disciplines such as physical chemistry. While this all makes sense for the natural sciences, the question is if such a level approach equally makes sense in the social sciences. There is something weird about the level approach which is well illustrated by Crawford and Novak (2018), when they introduced “sociological social psychology” which is according to these scholars different from “psychological social psychology.” This illustrates that the so-called interdisciplinarity in the social sciences seems to lead first and for all to more turfs and one can wonder if this helps integrate the different perspectives. Interestingly, the Crawford and Novak (2018) book is entitled “Individual and Society,” but in the subject index, there is no entry for “social theory.” Although anecdotal, this can be regarded as a marker for the barriers that exist between the social theorizing crowd and the scholars that focus on empirical research.

The second assumption of SWT is that persons and societies are two inseparable manifestations of how the human species has developed a non-genetical-driven mechanism to survive. The main difference between humans and non-human species is that in the latter, the “knowledge” to survive resides mostly in the genetic material of the species, while in the former, the “knowledge” to survive resides outside the body in a cultural environment from which human beings can appropriate it. Acknowledging this has consequences for the use of disciplinary perspectives in studying people and societies.

There is a long tradition of disciplinary divides in the social sciences that are taken for granted (Manicas, 1987; Van Langenhove, 2007). However, the social and psychological realm cannot be modeled in disciplines that each represent a layer such as is the case with the natural sciences. Nevertheless, persons are the subject of personality theories, and societies are studied in sociology and social theory. In reality, persons and societies are, as a result of the abovementioned transition from genetically determined behavior to culturally shaped behavior, so entangled that one cannot exist without the other. To emerge out of a process of natural evolution, there must be advantages to cultural evolution. And there also must be mechanisms to deal with the disadvantages. Furthermore, this shift from genes toward culture has brought with it a number of challenges. The first one is that the info to survive must have a valence to persons. This means that there are mechanisms needed that make people need to access the info available. Also, the info to survive is unevenly distributed. Some persons have access to info that others do not have. Therefore, there also needs to be systems that link persons to each other. One such system is the introduction of money as a way to objectify bartering.

The person–society entanglement is complex; it implies that the human mind needs to be regarded as something that extends beyond a single human brain and includes the interactions it is involved in. As rightly noted by Siegel (2011), the mind is both embodied and relational, and backed by the research he quotes, he claims that a brain cannot be disentangled from its interactions. This stands in sharp contrast with the traditional view that the human mind is a product of only the physical brain and its neurons, and not from its interactions.

#### Assumption three: the speech-act as a substance of the social and psychological realm

Personality and society are two sides of a mechanism that functions as a tool to maintain and develop a non-genetically based ecosystem that allows people and societies to cope with everyday situations. What binds persons and societies is speech-acts and conversations. Persons can utter speech-acts as part of conversations in which they perform the role of spokesperson for themselves. “I don't like this cheese. Can you pass me the salad bowl?” is an example of such a conversation. However, people can also utter speech acts on behalf of other entities that have personhood properties. “The court has decided that the defendant is found guilty” qualifies as such where a judge speaks on behalf of the law. So, people are not only taking part in conversations where they speak for themselves. A lot of conversations are between a “natural” person and an entity (or actor). “Do not smoke in this room” is a sign that reminds us of a conversation between members of parliament when they decided that smoking in public offices is no longer allowed. The sign “no smoking” can be compared with a broken record: it is a continuous repeat of a conversation that banned smoking and that now is used between the authorities who banned smoking and whoever enters a room where that rule applies. In other words, persons and societies are linked because both utter speech-acts in the course of conversations.

The third assumption of SWT is that within conversations, an extraordinary phenomenon exists: some words in a conversation can take the form of a speech-act. Such speech-acts have potentially enormous powers but the actual power depends on whether the speaker has the moral right to utter that speech-act and whether there are people who can take up that speech-act. One of the most dramatic claims in this regard is the following: “*Conversation is to be thought of as creating a social world just as causality generates a physical one*” (Harré, 1975, p. 65).

I follow Harré and other advocates of the discursive turn that the essence of the social reality is the ongoing stream of conversations in which speech-acts play a crucial role. Harré formulates it as follows: “the potent ‘things' in the human world are not people but the things they say” (Harré, 1990, p. 352). Indeed, speech-acts have enormous powers such as mobilizing people to do things. This implies that with speech acts one can act upon the material world without touching it. This means that by using speech-acts, persons can transcend space and even time. As illustrated by Van Langenhove (2020), persons have, for instance, the ability to—say—close a door, without exercising physical force on it, but by asking someone to do that. Similarly, speech acts can have effects over time (as in “when you leave, don't forget to close the door”).

In other words, it is speech-acts that for different reasons and with different mechanisms constitute and shape all sociological and psychological phenomena that make up the social realm. As a result, speech-acts can be seen as a mechanism to connect the brainpower of many individuals into a single collective power that outnumbers the capacity of individuals and outlives the life of the individuals.

Human beings are extraordinary in using language not only as a tool for communication but also as an instrument to create the social realm. Speech-acts have created structures that embed the life of persons. As stated by many scholars, the existence of society must therefore be related to our capacity for language. This is reflected in the by now long tradition of emphasizing the discursive aspects of the social and psychological realms. Social constructionism, social constructivism, the discursive turn, narratology, linguistic philosophy, and so on, all point to the importance of language in the ontology of the social realm as well as in researching it. However, it can be argued that conversations are the most fundamental aspect of the social reality, a point missed by many social constructionist scholars. The third assumption of the SWT, therefore, boils down to the idea that not written text but oral conversations should be at the heart of understanding society. The argument runs as follows:

A speech act in linguistics is an utterance that has a performative function in language and communication (Austin, 1962). Speech acts are commonly taken to include such acts as promising, ordering, greeting, warning, inviting, and congratulating. According to Searle (1995, 2009), there is at least one specific formal linguistic mechanism that acts as a single unifying principle that constitutes any institutional structure. That principle underlying the ontology of the social realm is the capacity of persons to “impose functions on objects and people where the objects and the people cannot perform the function solely in virtue of their physical structure” (Searle, 2009, p. 7). Searle calls these “status functions” as they imply a collectively recognized status. A piece of paper will count as a 20 EUR bill only if people give that status to that piece of paper. Status functions also carry what Searle calls “deontic power.” This is where the moral perspective comes in as deontic powers are all about “rights, duties, obligations, requirements, permissions, authorizations, entitlements, and so on” (Searle, 2009, p. 9). Deontic powers are, according to Searle, created by a specific sort of illocutionary speech-acts, namely “declarations.”

For many scholars who study language, the difference between spoken and written language is not really a point of concern. Even more, according to Ong (1989), there is a persistent tendency among scholars to think of writing as the basic form of language. We owe Ong to have pointed to some important differences between spoken and written language. For a start, an oral utterance vanishes as soon as it is uttered. A written sentence can be re-read many times and remains available as long as the carrier exists. Of course, what is spoken can be remembered, but words are occurrences and events that have no focus nor trajectory. The restriction of words to sound determines not only modes of expression but also thought processes. the oral utterance has vanished as soon as it is uttered. As a result, the relationship between oral expression and writing is asymmetric: oral expression can exist without writing, but writing is never without orality (Ong, 1989, p. 8). Writing makes “words” appear similar to things because we can think of words as visible marks signaling words to decoders. When an often-told story is not actually being told, all that exists is the potential in certain human beings to tell it. However, writing has not replaced orality: “the spoken word still resides and lives” (Ong, 1989, p. 8). For Ong, the main difference between speaking and writing is that the former allows to break down reality into various components while the latter allows for classifications and abstract explanations.

Seeing writing as the materialization of words into things has an important consequence, written words are residue. Oral tradition has no such deposit. All that exists is the potential that it is remembered by someone else and be retold. Of course, the text also needs a reader to come to life again. But when an oral story is forgotten, it has vanished. Furthermore, words when written become part of the visual world. In other words, writing brings the word into the realm of material artifacts, which gives it more possibilities for outreach.

Words are grounded in oral speech but writing locks them in the visual field. Spoken words are events that have no trace, and written words are materialized and become detached from the one who spoke them. They exist primarily in the Euclidian physical space and they enter the person/conversation grid when read by someone. On countless occasions, Harré has stressed the importance of conversations in his study of the philosophy of science, psychology, and social theory. His study contains several references to conversations as being the crucial element of social life and a model or metaphor for understanding specific elements of the social realm. For Harré “*the fundamental human reality is a conversation, effectively without beginning or end, to which, from time to time, individuals may take contributions*” (Harré, 1984, p. 20). Such a species-wide and history-long conversational web is regarded by Harré as the “primary” social reality. However, there is more to the primary social reality than people just talking. People do also things, they act, and the actions of people are to a large extent related to fulfilling tasks.

A formidable property of speech-acts is that they can have powers to influence both the social and the natural world! Imagine the author fancying a cup of coffee when writing this chapter. He can stop his writing and make coffee or he can ask his secretary to make him a cup of coffee. There are thus two ways of making coffee: one is by doing it (acting) and the other is by uttering a speech-act. But in the latter case, speech-acts alone will not do. When saying “Will you please make me a cup of coffee while I continue writing this chapter,” this will only result in coffee being served (a) if someone hears his speech-act and (b) if that person considers it appropriate to execute that demand. Or, in other words, one needs to have the proper moral rights to demand coffee. Exactly there lies a big difference between the natural and social world: the primary social reality does not exist independently of people and what happens and what can happen is subject to rules, conventions, rights, and duties.

## Developing the descriptive and explanatory capacity of SWT

Scientific theories serve two purposes: they provide a vocabulary to describe something observable, and they allow us to identify a hidden mechanism. A theory is then what connects the observable with the hidden mechanism. Hence, building a scientific theory of how societies and persons are connected should include three steps: (i) the introduction of a fine-tuned conceptual grammar to describe society and its relation to persons, (ii) the introduction of models that allow to postulate hidden mechanisms behind the descriptions, and (iii) the formulation of theories about how the hidden mechanisms relate to the observable. The ultimate challenge for SWT is thus to construct a lexicon for talking about the social realm that can be used for building theories about what we do not see. What follows can be considered as the first step in building such a theoretical corpus. Below this will be developed further by presenting the idea that societies are singular, the creation of an adequate grammar to describe the (singularity) of societies, and the introduction of an explanatory model to understand the way a society functions.

### The singularity of societies

According to Harré (1998, p. 1), there is a need for a “grammar” of a conceptual layout in the social sciences that would bring some clarity to the way we talk about persons, selves, or individuals. For Harré, the key issue is to develop a structured concept of personhood that recognizes that there is a personal singularity, meaning that no two people are alike, but still they bear many resemblances to one another. Individuality in this context means that one is a different thing from other things. Uniqueness on the other hand refers to being like no other things. Harré uses the example of a flock of birds to illustrate this. A flock of birds can, for instance, be made up of individual seagulls, but for an observer, they are interchangeable. Perhaps to a skilled observer, some individual marks can be observed. But it would be hard to identify a unique Jonathan Seagull in the flock. This tendency that all exemplars of a species are interchangeable increases as one descends on the ladder of organic beings. The lower on the ladder one gets, the less prominent individual marks are. Bacteria of a certain type will all behave in the same way, and there are no individualized bacteria that are unique and singular beings. This is so unlike human beings where uniqueness and personal singularity stand out when looking at the very diverse human behaviors that are deployed in coping with the many and very diverse situations a person encounters throughout the day. There is also some unity to detect. People construct autobiographies to express their unity across diverse situations. Still, according to Harré, this does not mean that the'self' is to be regarded as a kind of “entity” that sits somewhere inside a person or a mind. Rather, it should be seen as “a site from which a person perceives the world and a place from which to act” (Harré, 1998, p. 3). As such, the uniqueness of a person is the result of two features of personhood: the unique attributes of the position in time and space and the unique points of view related to what is perceived by the world around them. At any moment, people can be located in time and space as well as in one or several moral orders. That comes with a series of attributions and accounts. For instance, when assisting at a football game, a person can be identified as a fan of a certain team, which accounts for the cheering of that person when that team scores. But that same person can simultaneously also be a father, a university lecturer, a cigar smoker, a reader of David Lodge, and so on. This all adds up to being a unique person. For Harré (1998, p. 9), the self is an expression of singularity. It refers to two issues: on the one hand, there is the collection of attributes ascribed to the person, and on the other hand, it is the set of experiences one has encountered. The combination of these two means that there are no two persons with the same self.

The idea of the self is as a “site” where one reflects and where acts can be linked to the concept of society. It can be argued that society is a singularity as well. For a start, it is made up of all these unique persons as described above. But there is more: each of those persons has a unique perception of the world and of the society he/she is living in. That perception is limited much in the same way as the horizon limits what one can see of the world. There is also a societal horizon marked by the conversations one is participating too or aware of. Every person has only a very partial view of what happens in society, and that partial view is also unique. It starts from regarding one as a self, related to and surrounded by a circle of people with whom that person is occasionally engaging in conversations. Next comes a circle of indirect conversations and finally, there is a circle of people who stay outside the realm of direct or indirect conversations but of whom someone else has talked about. These are the second-order conversations. For instance, nobody who lives today has ever spoken to Napoleon and few will have read some of his writings. Still, a lot of people have some knowledge of the historical character that Napoleon was, because of “conversations” with history books or movies about his life.

Societies are made up of individuals, and while the individuals have a material body that makes one think of them as an entity, societies are not to be seen as a single entity. Hull (2013, p. 54) speaks of “the socially induced illusion of thinglikeness.” In staid, societies have to be regarded as a place in which human actions occur. Moreover, that “place” is full of resources that can or need to be used by people to act. Every situation a person encounters brings with it a series of potential positions that can be activated by the person or by what is happening in that situation (Harré and Van Langenhove, 1991). Suppose a person describes himself as a fan of a football club and that person is walking on the street, at that point, there is nothing that activates the football moral order. However, when a group of fans of a competing team approaches, this activates that order. In other words, persons are always in a position that can trigger moral orders to be active. And, just as the self is constructed by some of those actions, society is also born out of human acts. This means that both self and society are concepts that are used by people to frame themselves in time and space. The notion of “self” points to unity across time, while the notion of “society” refers to unity across situations. A person has one singular biography (or several versions of them) and lives in what appears to be one singular society of which s(he) is at the center. However, as that holds for all persons, one can say there is no singularity of societies but a society of singularities. Society is always a juxtaposition of many possible appearances. Both self and society need to be thought of as stories in which their uniqueness is both displayed and established. This resonates with Luhman's idea that the social world is not an object but a horizon that is inaccessible. As a result, Luhman proposes to replace the question of what society really is with the question of how society observes itself and is observed. This should, however, not lead to a de-ontologization of the world, as Luhman implies. Rather, it needs to be seen as an epistemologization of ontology.

### The sociocultural model by Harré and Vygotsky

**Figure 1:**
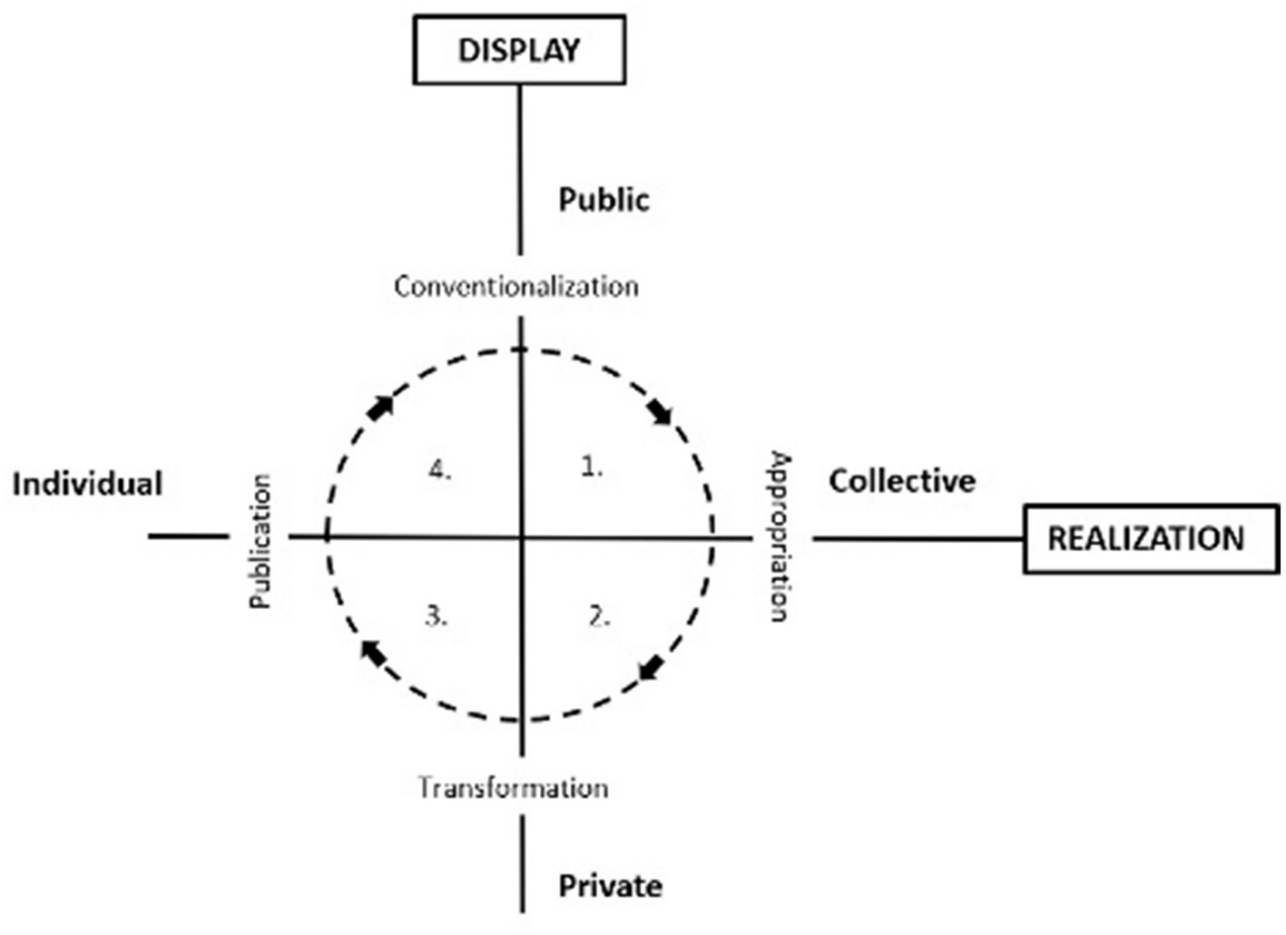
The Vygotsky model.

In what follows, a new look at societies is proposed that brings a new perspective to how society can be observed. It is based on the sociocultural model as proposed by Harré (1984) and that in turn is inspired by the study by Vygotsky (1976). Vygotsky's view on the relationship between psychological and social phenomena is rooted in his research on learning processes that boils down to addressing the dichotomy between individualistic and collectivistic approaches to knowledge generation and the dichotomy between private and public dimension manifestations of knowledge. In other words, there are two dimensions to consider: display and realization. The display dimension encompasses both public and private levels of knowledge presentation, where the public domain of the display dimension refers to the sharing of ideas. The private domain of the display dimension deals with the generation and modifications of knowledge in a non-public manner. For Vygotsky, these dimensions are not to be equated with each other. Rather, they should be regarded as orthogonal, one against the other, resulting in a matrix with four quadrants: public/collective, public/individual, private/collective, and private/individual. Vygotsky used this scheme to model the process of knowledge creation. This process involves four transitions from one to another cell: appropriation, transformation, publication, and conventionalization. Figure 1 presents the Vygotsky model as adapted by Harré (1984). The appropriation phase is about the transition from the public/collective quadrant. The transformation phase depicts the transition from the collective/private quadrant to the private/individual quadrant. The publication phase represents the transition from the private/individual quadrant to the individual/public quadrant. The conventionalization phase describes the transition from the individual–public quadrant to the public/collective quadrant.

The Vygotsky/Harré model offers a new look to societies by replacing the old opposition between (personal) agency and (societal) structure with a new conceptual distinction between four types of social realms in which social activities and processes and social entities as plotted on two dimensions (public/private and collective/individual). These two dimensions are inspired by the Vygotsky scheme as presented by Harré (1984) and it will be argued that this conceptualization exercise can be regarded as a major step forward in further developing the “Spoken World Theory” as introduced by Van Langenhove (2021). The two dimensions allow us to consider four distinct realms of social and psychological phenomena:

- the public/collective quadrant where one can locate all social phenomena that exist independently of a single person. Here, one can situate, for instance, social artifacts, social representations, general knowledge, and beliefs to be found in written texts as well as moral orders.
- the private/collective quadrant that contains elements from the public/collective quadrant that are appropriated by individual persons such as learned skills and opinions as well as a personal identity and personal worldviews.
- the public/individual quadrant that is filled with contributions from individual persons to the public sphere when people publicly act or talk. Both speaking and acting are to be regarded as performances that include both an expressive and a problem-solving part.
- Finally, there is the private/individual quadrant which consists of the cognitive phenomena that a person does not share with others such as inner talks and thoughts as well as intentions to act or talk.

The Vygotsky/Harré model can be used as a source model for describing society as consisting of four spaces situated at the crossing of two dimensions, a public/private axis and a collective/individual axis. It implies that society manifests itself in four ways: as conversations between persons, as social things created by persons, as worldviews of persons, and as intentions to act by persons. The conversations are public and individual, the realm of social things is collective and public, the worldviews are collective and individual, and the intentions of persons are private and individual. Moreover, the transitions between the four realms allow us to describe how society acts as an enabler of agency and how at the same time society is created by that agency.

Society can as such be regarded as four realms that organize the non-genetical aspects related to living one's life. The first realm is a worldwide and physical tangible set of social artifacts (things and persons) together with a set of intangible normative moral orders that can be appropriated by persons as individual umwelts in which persons have the power to formulate intentions that they can bring to the conversational space. In other words, society is a set of ongoing conversations in a landscape of social things and persons having the power to contribute to the conversations and develop a personalized worldview of society.

Thinking about the ontology of the social realm is not to be dissociated from epistemology. After all, a person observing a society can only do that from the point of view of someone who is inside that society. Therefore, by way of a thought experiment, one can imagine that a person observes the world, first by looking at it, then by listening to it, followed by reasoning and thinking about what (s)he saw and heard, and finally by acting in the world. Following the singularity approach, one can imagine that this person is the center of society, as any other person is the center of the social universe. Indeed, from a personal perspective, one always sees him or herself in the middle of a situation that is in turn part of a historical and diachronical series of events. While we always experience only a tiny bit of a societal phenomenon, that “here and now” experience can be linked to a tremendous amount of other things that with some imagination and endurance or perseverance at the end make through this loophole the whole society is involved. Let us assume our imagined person decides to go to the local football club stadium to watch a game of his favorite football team. That involves participating in an event that is part of society. As he is not alone in doing that, the experience is multiplied by thousands of others. The particular game can be related to the history of that club's performances as well as to the building of a stadium, the fabrication of sharps that some fans will wear, the mobilization of police forces and stewards to organize the crowd control, the brewing processes of the beers that the fans will drink, and so on. Everything that happens in a society is preceded by a myriad of conversations and actions that make that particular event possible. It will be followed by lots of other conversations about who performed well, if the referee was right regarding a penalty, and so on. In short, the singularity of a situation lies not only in the events *stricto sensu* but equally in the conversational reality that surrounds it.

What follows is a description of what can be experienced in the four quadrants of the Vygotsky scheme. We start with the public/collective realm where the visible things can be categorized. Next is the public/individual realm which consists of individual contributions to conversations and qualifies, therefore, as an audible realm. To this can be added the private/collective realm that involves the appropriated parts of the first two realms and finally the private/individual realm of intentions and readiness to act.

#### Seeing the social world

First, there is the visible realm. When a person looks around, he or she sees a physical and social world. People look at the physical world within a horizon that is before them as a circle with themselves in the center. From a physical perspective, it is a horizontal world dominated by one vertical power: gravity. The world in that observable plane consists of material things that belong to what is called nature (rocks, clouds, plants, animals, etc.). But there is also a social world that is fabricated by people and can therefore be called the world of social artifacts. This means that they are created by people and therefore have meaning to people. I see several types of social things that occupy this space: material objects created by humans and other persons, some of whom I know personally and others who are not familiar to me at all. This realm is the visible realm as it consists of all social things that have a material correlate, which does not mean that they are all in the same way visible.

The first category is the material objects created by people. That includes all kinds of material *artifacts* that would not exist without having been created by human beings and are therefore social. This includes cities, roads, landscaped gardens, Korean food, wine, books, sheets, and so on. The list is endless, and in today's world, almost everything on the whole planet is in a way social. A mountain is a purely natural phenomenon while a Terrill is the result of human mining and thus a social phenomenon.

The second category is persons. In a way, they are things too, as persons have bodies, but in contrast to all other sorts of things, they have the power of agency. We can see the bodies of other persons, and more importantly, we can talk to them, which allows us to influence them and to be influenced by them. More about that later.

The third category is the material objects that contain messages or information. Among them are written text as well as all other representations of information, or the carriers of data. They can be regarded as materializations of certain conversations.

The fourth category is the so-called institutional facts. Money, for instance, only exists because people agree that a banknote—which is in physical terms nothing more than a piece of paper—has, for instance, a value of 20 Euro.

Finally, there is a fifth category, the fields of influence, that is in itself not visible, but through the use of symbols or texts be made visible. When someone, for instance, crosses a road full of driving cars, that involves dealing with a field. Crossing is a physical act, but there is a social dimension to it as well. In Belgium, for instance, it is mandatory to drive on the right side of the street. That will not be indicated on every street, but people are supposed to know this and to be aware that other people expect you to drive on the right side of the road. There is a written text, the traffic law that prohibits one from driving on the left side. Another example is entering a house: in principle, one can enter every house, but some rules and regulations apply. Part of them are written down in legal texts that state that entering someone's other house is burgherly. The point is thus that amid the material artifacts that surround us, some artiefacts take the form of texts that divide the physical spaces in fields where all kinds of rules and regulations apply. One can think of these as fields that are linked to the written source as well as to the part of reality they apply to. Traffic laws, for instance, apply to roads, not to your backyard. So, people are surrounded by fields of moral orders that to a large extent limit their possibilities and determine what is appropriate to do or not and they have some knowledge about that (Van Langenhove, 2017).

#### Hearing the social world

The second way to perceive the social world is by listening. What people hear the whole day is people talking to other people, including themselves talking to other people. The social world can therefore be pictured as a worldwide and history-long web of conversations.

Conversations are the locus of speech-acts, that is words that can do or make do things. Such speech-acts have potentially enormous powers, but the actual power depends on whether there are people who are granted the power to utter that speech-act and people who take up that speech-act. It is speech-acts that for different reasons and with different mechanisms constitute and shape all sociological and psychological phenomena that make up the social realm.

As a result, speech-acts in conversations can be seen as a mechanism to connect the brainpower of many individuals into a single collective power that outnumbers the capacity of individuals and outlives the lives of the individuals.

Individual people are engaged in conversations with a limited set of other people. Next, there are conversations in which we did not participate, but we know about them because we heard about them. Finally, there are lots of conversations we know nothing about, some can reach us, but mostly they never affect us because they are beyond our conversational horizon. Although, we might use or encounter things that are products of some of those far-away conversations.

As such, the constant flux of conversations serves as a kind of glue between people. A crucial aspect of social reality is that it connects people through all kinds of conversations where information is shared. But conversations are also leading to speech-acts and this is a crucial aspect of the social reality as it creates a social world that becomes independent of the ongoing conversational reality. Symbolically, this can be called the audible realm as it consists mainly of what people say to each other. The building where I write this essay is not a university building but a priory. It becomes a university as a result of the activities performed in it.

People talking to each other can be regarded as the most important aspect of the observable social realm. It is through talking that people are connected to other people. There is, for instance, a chain of conversations between myself and those involved in designing and manufacturing my PC. Probably thousands of conversations have been taking place starting from the conversation between the guy who decided to fabricate this type of PC and the investors he persuaded to the one guy at the counter of the shop to whom I paid when purchasing this PC and with whom I did have a conversation. Not only is this chain of conversations connecting people, but also it makes it possible that things happen that need to happen if one wants to build and sell a PC. But the main feature of this is that I do not have to know how to design a PC to use it (Sloman and Fernbach, 2017). Therefore, my brain is not the only tool I use for doing things, I have access to the brainpower of many others. The instrument for this is the speech-act that requests others to do something. For long, the only actors that could produce speech-acts were people. Today we are also told to do things by machines as well. Think of the navigation system in your car that tells you when to turn right…

Everyday life can best be thought of as the directly experienced everyday conversations of people together with the material substances involved in the actions and conversations (other people, books, traffic signs, and so on). It is the daily flow of conversations in which people engage and the social agents and artifacts that surround people. But next to that direct experience, people can also experience conversations in which they did not take part. Take, for instance, the conversation that the author just had with his wife while writing this text. This is in principle a possible experience for the reader of this chapter as well, although that conversation took place in the past and although you were not there. To experience that conversation, the author could write a transcript or account of what he and his wife just discussed. But as the author will not do this, this part of the social reality remains unexperienced for the reader.

Together, artifacts, people, and conversations form the observable social umwelt in which persons operate. All three categories do have a material substance that allows us to perceive them through our senses. Moreover, as material things, they can all be situated in space and time. It is observable that the social realm has been studied by many different social science disciplines according to a division of labor. The conversations are the providence of linguistic and communication studies. Artifacts are studied by disciplines such as cultural studies (art), economy (money and factories), sociology (institutions), and law (the judicial system). Persons are the subject of psychology. But the link between the object of study and the discipline is never straightforward and there are enduring debates on how the different disciplines relate to each other.

Social reality can be experienced directly only through talk or by seeing things that are the products of speech-acts. The “social” and the “psychological” cannot be seen as such. As Searle (1995, p. 4) noted “the complex structure of social reality is, so to speak, weightless and invisible.” It can only be talked about, and it is in talk that the social is constituted. Of course, the material substance associated with social phenomena can be seen: we can see the buildings of a university, but we never can “see” the university as a functioning social entity without being involved in some sort of conversation. We can see tears, but we cannot understand why somebody is grieving without some spoken information. We can see a castle, but we can never experience the medieval social organization of life within the castle as there is no one from the Middle Ages still alive.

Most of the ongoing conversations between people are passing exchanges that somehow disappear soon after they take place. For instance: the author's wife just asked if he would like a cup of coffee. If it were not for having used this here as an example, the author would probably have soon forgotten about this trivial event. And if he had not written about it, this little social event would have remained un-experienceable for others. Most of what people tell each other in conversations are utterances that after being uttered, immediately “die” and disappear. In some cases, as is, for instance, the case with academics when they write a book, people hope that what they say or write will live a longer life. The academic adagio “publish or perish” testifies to that.

If one accepts that persons, artifacts, and conversations are the three observable elements of social reality, the next question is whether this is an exhaustive description of what the social realm is. Let us again look at the material world: with our senses, we can observe a good chunk of it, but we know there is more. Radio waves, for instance, are a particular part of the physical reality and they surround us at all times, but we cannot perceive them unless we have a radio that transforms the radio signals into sound. It can be argued that the same holds true for social reality: there are social fields that are as such not visible, but they are there and they have a profound impact on what people do and on how a society functions.

A good way to explore the power of the metaphor is to imagine that fields surround us at all times and infuse what people do. As Musser puts it: “We are swimming in it and it is always tugging upon us. We never see it directly, but it makes its presence felt by communicating forces from one place to another” (Musser, 2015, p. 72).

#### Imagining the social world

The third way to experience the social world is by combining the above experiences with thinking about it and thus constructing a personal umwelt that acts as a horizon within the horizon. For instance, one might walk by a prison. But most people have no idea what goes on in a prison and can hardly imagine what it is to be imprisoned. But one can read about it, watch TV documentaries, perhaps talk to people who work in a prison, write letters to inmates, etc. Furthermore, what holds for prisons also holds for all other aspects of the social reality in the public domain.

While the first two realms span the whole globe and cover the whole of history, they cannot be experienced by a single person in their totality. What people see and know about society is always limited to personal experience with additional information or knowledge of things that that person was not experiencing directly. This results in a personal umwelt of knowledge and beliefs about the world that can be labeled the worldview.

#### Shaping the social world

Finally, there is a fourth way of experiencing the social world that begins with intentions and leads to participating in conversations as well as acting in the realm of social things.

The relation between persons and society is not that persons are passively undergoing the societal activities that they hear and see when they fall in their horizons. No, people can also contribute to society, by participating in conversations, producing social artifacts, raising kids that become new persons, and on top of it, they cannot only do those things but can also reflect upon them and share the reflections in conversations. In sum, by showing agency people shape society.

At this point, I want to advance the claim that a society is a singularity created by speech acts in the four quadrants of the Vygotskian space and that the conservation of a societal order as well as its changes are driven by speech acts that move from one quadrant to another. Let us illustrate this with an example. Suppose a person has the intention to write a book, that intention can be situated in the private/individual realm as a plan that someday will be executed. When that person starts talking to a publisher, signs a contract, and begins writing, the writing process of a book becomes part of the conversational realm that is public and individual. Once the book is published, it becomes part of the public/collective realm and when a reader starts reading it, the experience of reading that book is for that reader a private/collective experience. And perhaps that experience will lead to the reader dreaming of writing another book...

Together, these four realms make up the totality of a society. It can be argued that the most important realm is that of conversations as it is through conversations that all other social phenomena have their origin in conversations and therefore only exist because of the conversations. Because of the fluid nature of conversations, one can picture parts of society as only existing at a particular point in time. If remembered, that part can exist for the lifetime of that person, if recorded, that particular art of society can outlive the persons involved. In that sense, both personality and society are two mechanisms that allow the individuals of the human species to complement the genetic basis of survival with a system of cultural resources that can be used for coping with everyday reality in both an individual and collective way.

## Conclusion

The essence of the SWT is the idea that persons and societies have to be seen as one reality, because on the one hand, persons cannot exist without a society that surrounds them, and on the other hand, societies cannot exist without persons who produce speech-acts which are the constituents of society. The idea of considering persons and societies as one analytical reality may seem odd because we perceive persons as linked to bodies of human beings, which are for sure distinct physical entities, and societies are often related to structures, but the institutions that embody the structures are visible as well.

This article presented a brief outline of a vocabulary that can be applied when analyzing every social phenomenon possible by identifying what happens in each Vygotskian quadrant and by mapping how via speech-acts the phenomenon moves around the four Vygotskian spaces. Together, they can be regarded as a personsociety, a neologism that is inspired by the Einsteinian notion of timespace. Metaphorically, the old duality between persons and structures can be compared to the relationship between time and space. In the Newtonian world, time and space are independent of each other and together they form an Euclidian space in which matter can be situated on the Y and X axes of time and space. It took the genius of Einstein to advance the idea that on a different scale, space and time are not independent. Hence, since Einstein generally accepted the concept of timespace that does not contradict the Newtonian worldview, it only limits its validity to a specific magnitude. Reality at the quantum level and the astronomical scale are Einsteinian, in between is the Newtonian reality as experienced in daily life. Taking this as a metaphor allows us to think of the social world as a realm that is also non-Euclidean and where it is impossible to understand the functioning of either persons or society without seeing them as one reality. In such a view, the social world equivalent of matter as situated in a time and space dimension are speech-acts that are situated on two non-Euclidean dimensions: a conversational one made up of a network of speech-acts and a dimension of agents that produce speech-acts that have all kind of effects (see Harré, 1987, 1988, 1997).

Vygotsky has famously stated that the mind is social. The SWT supports this insight, but also that the reverse holds as well: society is psychological. Society also binds persons to a social environment to complement their genetic material with cultural resources that are needed to cope with the situations that they encounter. At the core of this theory is the postulate that everything social or psychological can be situated in four conceptual spaces: the realm of conversations, the realm of visually observable artifacts and not directly experienceable institutional facts and fields, the realm of appropriated umwelts, and the realm of intentions. Together, these four spaces are ontologically from a social and psychological point of view.

The above-presented ontological grammar has several implications that can be further analyzed and developed. The first one is that social theorists should stop regarding society as a thinglike entity. A society does produce social artifacts, but it is itself not an artifact. Rather, it can be compared to icons that exist as images of the reality in which persons cope with the situation they are in (Harré, 1975). As such, there are as many images of society as there are persons.

Furthermore, the realm of social things (including texts and persons) serves as a toolbox of fields for persons. Some of the artifacts can be regarded as fields and so do certain situations. The most important one seems to be the moral orders which influence what is appropriate behavior. The personsociety entity is driven by two things: enabling the agency of persons to make society function and the containment of agency to hold persons and society together. The tool to realize that is the speech-act in conversations. According to the SWT, conversations are the locus where society is created, they are the locus where mind and society connect, they are the locus where persons can enlarge their personal capacities, and finally they are the locus where society operates as an enabler and container of personal agency.

The SWT as partly outlined above, builds on three insights from Rom Harré. First, it takes conversations as the primary reality of society. In Harré's words: “there is a species wide and history-long conversation, only partially available to individual human beings, as their social Umwelten” (Harré, 1990, p. 350). Second, it sees speech-acts as the fundamental entity by which the social and psychological realm is created and changed. For Harré, this implies that there are only two powerful entities in the universe: elementary particles and persons! Third, it treats the social realm as a moral space, rather than as a realm of causes and consequences. This has enormous consequences for rethinking and reorganizing SSH research. As Harré asserts, the challenge is to replace the causal frame with normative readings: “Perhaps there is enough material locked up in technically opaque rhetorics to fuel a grand undertaking, the recovery of a scientific understanding of important aspects of our culture. That would indeed be something worth doing.” (Harré, 2002, p. 1, 188).

Together, these three insights call for a new look at how SSH can play a role in changing the social realm. According to Harré, the reconstruction of society can happen at any time in any conversation. This means that formulating new theories about the social realm is a road that can lead to societal evolution. Says Harré: “it is not their truth or falsity that is of importance but their role as guides of action” (Harré, 1990, p. 303). This echoes Marx in his 11th thesis on Feuerbach, where he called philosophers to not only interpret the world but to change it. However, the power of social theory speech-acts depends upon to what extent they are taken up in the different storylines of society, as well as on the status of that social theory. One can only hope that more theories will emerge that fulfill the conditions of sound research and that they will be convincing enough to challenge simplistic or wrong ideas about the social realm.

## Data availability statement

The original contributions presented in the study are included in the article/supplementary material, further inquiries can be directed to the corresponding author.

## Author contributions

The author confirms being the sole contributor of this work and has approved it for publication.

## Footnotes

^1^https://en.wikipedia.org/wiki/Society retrieved on 24/09/2023.

^2^See for instance Deutsch (1998), Rovelli (2016), and Carroll (2020).

^3^For overviews of the research of Harré, see Bhaskar (1990),Van Langenhove (2010), and Christensen (2019).

^4^The terminology for talking about persons is straithforward: personhood is what distinguishes a person from a non-person, and every person is said to ‘have' a personality, that is an individualized singular personal identity. Th eterminology for talking about societies is less developed, but one could formulate something similar: sociohood is what distinguishes a society from a non-society, and every society is said to have sociality, that is an individualized singulars manifestation.

^5^This claim is empirically backed by De Waele and Harré (1976) and Harré and Erneling (2012).
